# 

**Published:** 1883-01

**Authors:** 


					﻿List of Original Contributors to Volume IV.
ABBOTT, FRANK...........................M. D.
ATKINSON, W. H................... M.	D., D. D. S.
BARRETT, W. C...............M.	D„ D. D. S., M. D. S.
BLOUNT, A. A......................M.	D., D. D. S.
BODECKER, C. F. W...............D.	D. S., M. D. S.
BOGUE, E. A...’...................M.	D., D. D. S.
BROWN, G. CARLETON....................D.	D. S.
BROWNING, CLAUDE........................M. D.
BUNTING, E. H...........................DR.
CALLAN, PETER A.........................M. D.
CLARK, F. Y. .....................M.	D„ D. D. S.
CLOWES, J. W..........................D.	D. S.
COTTRELL, S. PARKER ....................M. D.
DARBY, EDWIN T.... ...............M.	D., D. D. S.
FRANCIS, CHARLES E..............D.	D. S., M. D. S.
GIBNEY, VOLNEY B........................M. D.
HARLAN, A. W.......................... D.	D. S.
HAYHURST, J.........................  D.	D. S.
HOWE, J. MORGAN ..................M.	D., M. D. S.
HOWLAND, E. P.........................D.	D. S.
HUNT, LEIGH H...........................M. D.
KINGSLEY, N. W..................D.	D. S., M. D. S.
LINE, J. EDW..........................D.	D. S.
LOOMIS, ALFRED L...................A.	M., M. D.
MILLER, W. D......................M.	D., D. D. S.
OSMUN, J. ALLEN.........................DR.
OTIS, FESSENDEN N.......................M. D.
PECK, EDWARD S......................M.	A., M. D.
PERINE, GEO. H........................D.	D. S.
REQUA, JOSEPH............................DR.
RICHARDS, W. P...........................DR.
ROBINSON, J. A........................D.	D. S.
ROSENTHAL, ED ..........................M. D.
ROSS, A. M............................D.	D. S.
STOCKWELL, C. T.......................D.	D. S.
SYMS, PARKER............................M. D.
TEES, AMBLER........................  D.	D, S.
VAN MARTER, J. G................. A.	B., D. D. S.
TRUEMAN, W. H........................  D.	D. S.
WALES, PHILIP S.........................M. D.
WENNING, W. H...........................M. D.
WHITTAKER, JAS. T.......................M. D.
Contributors:
ALF. L. LOOMIS, M.D.,.........New	York.
FESSENDEN N. OTIS, M.D.......
F. H. BOSWORTH. M.D..........
J. L. LITTLE. M.D.............
J. WILLSTON WRIGHT, M.D......
C. E. FRANCIS, D.D.S., M.D.S..
A. J. C. SKENE, M.D.....Brooklyn,	N. Y.
C. A. MARYIN, D.D.S......
EDWIN T. DARBY, M.D., D.D.S...Phil., Pa.
P. S. WALES, M.D...Surg.-Gen’l U. S. Navy.
H. F. CAMPBELL, M.D.........Augusta,Ga.
C. H. MASTIN, M.D., L.L.D....Mobile, Ala.
J. J. CHISOLM, M. D...Baltimore,	Md.
C. F. BEVAN, M.D........
I.	E. ATKINSON, M.D.....
II. McGUIRE, M.D.......Richmond,	Va.
F. P. PORCHER. M.D....Charleston, S. C.
JAS. T. WHITTAKER, M.D., Cincinnati, 0.
J.	RANSOHOFF, M.D., F.R.C.S.
F. FORCHHEIMER, M.D.....
A. R. JACKSON, M.D......Chicago,	Ill.
S. V. CLEVENGER, M.D......
F.. F. INGALS^M.D.........
J. H. CARSTBWS, M.D.....Detroit, Mich.
PROSPECTUS
For Vol. IV, 1883, of the Independent
Practitioner.
This Journal is independent of colleges,
cliques, isms, or any advertising firm ; there-
fore impartiality pervades its columns in the
sole interest of the Profession.
It is cosmopolitan in its circulation and
scientific resources ; consequently of equal
interest to the specialist and general practi-
tioner everywhere.
That which is new and useful in Medicine,
Surgery, Obstetrics, Dentistry, Pathology and
Popular Science is given to its readers in an
intelligent and concise style.
				

